# Evaluation of novel biomaterials for cartilage regeneration based on gelatin methacryloyl interpenetrated with extractive chondroitin sulfate or unsulfated biotechnological chondroitin

**DOI:** 10.1002/jbm.a.37364

**Published:** 2022-01-28

**Authors:** Valentina Vassallo, Anastasia Tsianaka, Nicola Alessio, Jana Grübel, Marcella Cammarota, Günter E. M. Tovar, Alexander Southan, Chiara Schiraldi

**Affiliations:** ^1^ Department of Experimental Medicine, Section of Biotechnology, Medical Histology and Molecular Biology University of Campania “Luigi Vanvitelli” Naples; ^2^ Institute of Interfacial Process Engineering and Plasma Technology IGVP University of Stuttgart Stuttgart Germany; ^3^ Fraunhofer Institute of Interfacial Engineering and Biotechnology IGB Stuttgart Germany

**Keywords:** cartilage regeneration, chondroitin, extracellular matrix, gelatin‐methacryloyl, MSC differentiation, scaffold

## Abstract

Gelatin is widely proposed as scaffold for cartilage tissue regeneration due to its high similarities to the extracellular matrix. However, poor mechanical properties and high sensitivity to enzymatic degradation encouraged the scientific community to develop strategies to obtain better performing hydrogels. Gelatin networks, specifically gelatin‐methacryloyl (GM), have been coupled to hyaluronan or chondroitin sulfate (CS). In this study, we evaluated the biophysical properties of an innovative photocross‐linked hydrogel based on GM with the addition of CS or a new unsulfated biotechnological chondroitin (BC). Biophysical, mechanical, and biochemical characterization have been assessed to compare GM hydrogels to the chondroitin containing networks. Moreover, mesenchymal stem cells (MSCs) were seeded on these biomaterials in order to evaluate the differentiation toward the chondrocyte phenotype in 21 days. Rheological characterization showed that both CS and BC increased the stiffness (*G'* was about 2‐fold), providing a stronger rigid matrix, with respect to GM alone. The biological tests confirmed the onset of MSCs differentiation process starting from 14 days of in vitro culture. In particular, the combination GM + BC resulted to be more effective than GM + CS in the up‐regulation of key genes such as collagen type 2A1 (COLII), SOX‐9, and aggrecan). In addition, the scanning microscope analyses revealed the cellular adhesion on materials and production of extracellular vesicles. Immunofluorescence staining confirmed an increase of COLII in presence of both chondroitins. Finally, the outcomes suggest that BC entangled within cross‐linked GM matrix may represent a promising new biomaterial with potential applications in cartilage regeneration.

## Graphical abstract

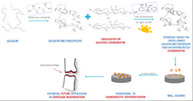

**Abbreviations**
AGN: aggrecan
BC: biotechnological chondroitin
CS: chondrotin sulfate
DMEM: Dulbecco's‐modified Eagle's medium
ECM: extracellular matrix
FBS: fetal bovine serum
GAGs: glycosaminoglycans
GM: gelatin‐methacryloyl
HA: hyaluronic acid
LAP: photoinitiator lithium phenyl‐2,4,6‐trimethylbenzoylphosphinate
MAAnh: methacrylic anhydride
MSCs: mesenchymal stem cells
OA: osteoarthtritis
PBS: phosphate buffered saline without MgCl_2_ and CaCl_2_

## 1 INTRODUCTION

Biopolymers have an important relevance for design of functional scaffolds, providing three‐dimensional (3D) structures able to support the cellular adhesion, growth, proliferation, and even differentiation.^1^ Bioscaffolds can be based on natural polymers such as collagen, gelatin, alginate, chitosan, hyaluronic acid (HA), chondroitin sulfate (CS) or synthetic polymers, for example poly(ethylene glycol).^2^, ^3^ During the last decades, engineered scaffolds gained importance thanks to their ability to promote vascularization, tissue formation and/or regeneration.^4^ Gelatin is a water‐soluble collagen‐derived biopolymer and it has been considered suitable for 3D architecture in cell culture models and tissue engineering applications because it is biodegradable, biocompatible, and bioactive.^5^ Despite the numerous advantages of gelatin use, poor mechanical properties, fast enzymatic degradation, and low solubility in concentrated aqueous media represent potential issues for its applications.^6^, ^7^ For these reasons, gelatin is commonly chemically modified; in particular, it is functionalized with methacryl groups (gelatin‐methacryloyl [GM]) and cross‐linked through photo‐induced reaction as previously introduced by van den Bulcke et al. in 2000.^8^ Often, modified gelatin is coupled to other glycosaminoglycans (GAGs) such as CS or HA^9^, ^10^, ^11^ to better mimic the extracellular matrix (ECM). The mechanical properties of resulting GM hydrogels and their biocompatibility are important to well resemble and substitute specific tissues such as bone,^12^, ^13^ cartilage,^14^, ^15^ adipose tissue,^16^ and cardiac tissue.^17^ However, a relevant aspect related to cross‐linking procedures is their potential toxicity for the cells, due to release of unnatural molecules possibly difficult to metabolize and thus accumulating in the cells maybe interfering with the cell cycle. Thus, cytocompatibility and cytotoxicity have always to be assessed before any attempt toward tissue engineering with chemically modified and/or unnatural polymers. In fact, sometimes the chemical modification of gelatin and/or of GAGs coupled to it can reduce the biological function. In this respect, La Gatta et al. 2021^18^ tested an enzymatically modified gelatin interpenetrated with unmodified HA alone and/or in combination with a biotechnological chondroitin (BC) for bone regeneration. This latter study showed that the produced hydrogels were not cytotoxic but, on the contrary, prompted the osteogenic differentiation highlighting the effectiveness of unmodified GAGs combined with modified gelatin.^18^ BC has been obtained through a patented biofermentative production process^19^ and its potential introduction in medical applications could represent an alternative to extractive CS and overcome diverse arising limitations (e.g., contaminations during the purification steps and ethical and religious issues).^20^ Hence, given the microbial capsular polysaccharides similarity to chondroitin, biotechnological processes exploiting specific *Escherichia coli* (*E. coli*) *strains* and tailored downstream procedures may be a potential nonanimal sources of this GAG.^21^ Most of the extractive CS used for medical purposes is obtained from terrestrial and marine animal sources and present different biochemical features in particular in term of molecular weight and sulfation patterns.^22^, ^23^ Scientific evidences displayed that the sulfation grade is responsible for a particular biological activity and marine CS has sulfation patterns different from those of terrestrial CS.^23^ Given that BC and marine CS are more similar with respect to molecular weight compared to terrestrial CS but, different for the absence of sulfate groups, both of them were here employed to compare their bioactivity, when embedded in a cross‐linked gelatin matrix.

Currently, damage and loss of cartilage caused by trauma, sports‐related erosion, chronic pathology as arthritis can cause disability among adults. In addition, untreated cartilage defects can evolve into osteoarthritis (OA) and thus, an effective treatment for cartilage injury should be developed.^24^ In this context, stem cells represent a potential solution for cartilage regeneration^25^ and several studies have been focused on the application of different types of stem cell sources such as, mesenchymal stem cells (MSCs),^26^ umbilical cord‐derived stem cells^27^ and adipose derived stem cells.^28^ MSCs are a subpopulation of mesenchymal stromal cells existent in the stroma of numerous organs and tissues, mostly in bone marrow and adipose tissue.^29^ The stem cells present in MSC cultures are able to differentiate into chondrocytes, osteocytes, adipocytes, and prompt tissue regeneration.^30^ These characteristics make the MSCs particularly interesting for cartilage tissue engineering.^24^

Latest scientific literature reports that GM promotes MSCs differentiation into chondrocytes,^24^ the goal of this set‐up was to investigate whether the timing of differentiation and/or the properties/composition of the ECM produced may be improved in presence of extractive sulfated chondroitin (CS) and/or not sulfated BC. Specifically, CS is a natural polysaccharide composed of D‐glucuronic and N‐acetyl‐D‐galactosamine disaccharide units and it is well known to have copious biological effects, such as anti‐inflammatory activity and support of cellular growth.^20^, ^31^ On the other hand, as previously explained, BC has been obtained through a patented biotechnological process.^19^, ^32^ Recent studies proved BC to be more effective compared to CS in counteracting the OA related inflammatory processes and modulating the secretome profile.^33^ In addition, a previous work demonstrated that BC was able to maintain the in vitro human derived nasal chondrocytes phenotype for a longer time period in comparison to CS.^34^ Since then, other cell models were established in our laboratories and the functionality of BC was notably superior on some aspects to sole HA or CS (e.g., OA chondrocytes and synoviocytes harvested and sorted from surgical removed joints).^33^, ^35^ Very recently, Alessio et al. 2021 reported that hyaluronic acid combined to CS or BC, additioned to the media in a late phase of culture (terminal differentiation) improved chondrocytes differentiation.^36^ For these reasons, this experimental work was focused on the development of 3D scaffolds based on the well‐known GM, photo‐cross‐linked in presence of CS or BC, and compared to the sole cross‐linked GM‐based scaffold. An extensive mechanical and chemical analysis was performed, and then the scaffolds were seeded with MSCs, to evaluate cartilage tissue engineering, identifying specific biomarkers and cell/construct morphological changes after 7, 14, and 21 days of in vitro culture.

## 2 MATERIALS AND METHODS

### 2.1 Materials

The following materials were purchased from Sigma‐Aldrich (Germany): Dulbecco's phosphate buffered saline without MgCl_2_ and CaCl_2_ (PBS^−^), methacrylic anhydride (MAAnh) and the photoinitiator Lithium phenyl‐2,4,6‐trimethylbenzoylphosphinate (LAP). For the gelatin synthesis, scaffolds production, yield, swelling and rheological studies the used PBS^−^ was self‐made with 137 mM NaCl, 2.7 mM KCl, 1.5 mM KH_2_PO_4_, 8.1 mM Na_2_HPO_4_·2 H_2_O, pH 7.4. Gelatin Type B (limed bovine bone, 232 g Bloom, viscosity: 2.8 mPa·s) was donated by Gelita (Germany) and Macherey‐Nagel filter paper (grade MN 614 1/4) was obtained from Carl Roth (Germany). Dialysis membranes (molecular weight cut‐off: 12–14 kDa) were purchased from Medicell International Ltd. (UK). Sodium 3‐trimethylsilyl‐propionate‐2,2,3,3‐d4 (TMSP) was bought from Merck (Germany). BC (MW; 35 ± 3 kDa, purity of 95 ± 5%, EU/mg <0.05) was produced through a patented fermentation and purification process using the strain *E. coli* RfaH O5:K4:H4 following specific protocols.^32^, ^37^ CS (MW; 30 ± 3 kDa, purity of 95 ± 5%, 0.1 EU/mg) was provided by IBSA and was extracted from shark fins/cartilage.

### 2.2 Synthesis of gelatin derivatives

GM derivatives were prepared with tenfold (GM10) molar excess of MAAnh with respect to a nominal amino group content of 0.35 mmol·g^−1^ as previously reported.^5^ Briefly, gelatin (25.06 g) was dissolved in deionized water (250 ml) at 40°C and its pH was adjusted to 7.25 using an automatic titration device. Within 30 min, 14.66 g of MAAnh were added. The reaction mixture was strongly stirred for 5 h, keeping its pH at 7.25. Then, the reaction mixture was filtrated and its pH was adjusted to 9.5. After leaving the mixture at 4°C for 2 days, the solution was dialyzed for 4 days against deionized water at room temperature. Afterwards, the pH was adjusted to 8.5 and the solution was freeze‐dried. The methacryloylation process was confirmed by ^1^H‐NMR spectroscopy according to Claaßen et al. 2018.^38^ Briefly, modified gelatin was dissolved in D_2_O and sodium 3‐trimethylsilyl‐propionate‐2,2,3,3‐d4 (TMSP) was used as internal standard. The degree of methacryloylation (DM) was calculated through ^1^H‐NMR spectra from a Bruker Avance 500 spectrometer (500 MHz, room temperature).

### 2.3 Preparation of GM10‐based hydrogels

All components of the hydrogel formulations were measured gravimetrically. Specifically, hydrogels containing 10% (wt/wt) GM10, 10% (wt/wt) GM10 with 2.5% (wt/wt) BC, or 10% (wt/wt) GM10 with 2.5% (wt/wt) CS were prepared by mixing the biopolymers and subsequent photo‐initiated radical cross‐linking as described below. In detail, GM10, BC, and CS were separately dissolved in PBS^−^ for 2 h at room temperature. When the powders were completely dissolved, BC or CS solutions were added to GM10 solutions and the obtained solutions were stirred at room temperature for 1 h. Then, the LAP was added to the hydrogel formulations in order to have 0.5% (wt/wt) of LAP with regard to GM10. The formulations were briefly vortexed at room temperature. Afterwards, the solutions were poured into a cylindrical mold (30 mm diameter, 1 mm depth) and covered by a quartz glass pane removing the air. Then, chemical cross‐linking was done by exposure to UV light (>300 nm with an emission maximum around approx. 365 nm, 50 mW·cm^−2^, sol2, Dr. Hönle AG) for 15 min. After curing, the quartz glass pane was removed and the cross‐linked hydrogels were taken out of the mold and washed in PBS^−^.

### 2.4 Hydrogel yield and swelling studies

Hydrogel samples were weighed for their initial masses *m*(start) immediately after cross‐linking and washed in ultrapure water or PBS^−^ at 25°C for 3 days changing the washing medium every 24 h. After these days, the swollen gels were weighed for the swollen masses *m*(swollen), vacuum‐dried over‐night (VDL 53, Binder GmbH), and weighed again for their dry masses *m*(dried). The equilibrium degrees of swelling (*EDS*) of the washed hydrogels were determined using *m*(swollen) and *m*(dried) by the following equation:

$$\mathrm{EDS}=\frac{m\left(\text{swollen}\right)\;}{m\left(\text{dried}\right)}\cdot 100\%.$$

Moreover, hydrogel yields (*y*) were calculated through the following equation, using the initial total polymer mass fraction *β*(polymers), the sum of GM10, BC, and CS mass fractions:

$$\;y=\frac{m\left(\text{dried}\right)\;}{\beta \left(\text{polymers}\right)\cdot m\left(\text{start}\right)}.$$

The measurements were performed in triplicates on three independent experiments and the results presented as mean ± SD.

### 2.5 BC and CS release studies

In order to verify BC and CS release from scaffolds, specific analyses were performed using a capillary electrophoresis HPCE instrument (P/ACE MDQ, Absciex, USA), equipped with a deuterium lamp and a photo diode array detector, with an un‐coated fused‐silica capillary (50 μm I.D., 70 cm of total length, 60 cm of effective length, Absciex, USA). Specifically, about 1 ml of PBS^−^ withdrawn from washes of hydrogels was ultrafiltered using 30‐kDa polyethersulfone membrane and the permeate fractions were analyzed. The feed flow rate and TMP were kept constant at 1 ml/min and 0.3–0.6 bar, respectively, during the process. Ultrapure water (MilliQ; Millipore, USA) was used as DF buffer. At each experimental analyzed time point, the amount of BC and CS released (%) was quantified following this equation:

$$\mathrm{GAG}\;\text{released}\;\left(\%\right)=\frac{\left(\mathrm{GAG}\;\text{released in}\;\mathrm{PBS}\;\right)}{\left(\mathrm{GAG}\;\text{content in the starting biomatrial}\right)}\cdot 100\%.$$

### 2.6 Rheological characterization of hydrogels

After the cross‐linking, the hydrogels were washed in PBS^−^ at 25°C over‐night. The oscillatory dynamic measurements were performed with a Physica Modular Compact MCR301 Rheometer from Anton Paar (Germany) using a parallel‐plate model (8 mm, 25°C, 0.16 N load). Swollen cylindrical samples were punched with a 8 mm hole punch and oscillatory strain amplitude sweeps (0.01% ≤ γ ≤ 100%) were performed at a frequency of 1 Hz. Moreover, the storage (*G'*) and loss (*G″*) modulus were evaluated also as function of the frequency (0.1 ≤ Hz ≤ 100) at γ 0.1%. Data are reported as average of three independent preparations ± SD.

### 2.7 Hydrogel stability toward hydrolysis and enzymatic degradation

Stability to hydrolysis under physiological conditions was explored by leaving the hydrogels to soak in cell culture medium at 37°C. The mass losses (%) after 7, 14, and 21 days were calculated with respect to the dry starting mass. Moreover, materials stability to collagenase action was also evaluated incubating them into a collagenase solution (3 U/ml) (Collagenase Type I, Gibco, USA) in culture medium at 37°C, and the mass loss (%) again was calculated with respect to the dry starting mass after 3 and 16 h of enzymatic digestion.

### 2.8 MSC seeding and growth on GM10‐based hydrogels to induce chondrogenic differentiation

MSCs were kindly provided by the research group of Prof. Galderisi of the Department Experimental Medicine of University of Campania “Luigi Vanvitelli.” Chondrogenic differentiation was performed in GM10 based scaffolds. Briefly, the dried cross‐linked biomaterials were placed in a standard 24‐well culture plate, 5.0 × 10^4^ MSCs aliquots were suspended in 10 μl of chondrogenic culture medium composed by: DMEM (Dulbecco's‐modified Eagle's medium, Gibco, Invitrogen) with 10% vol/vol of FBS (Fetal Bovine Serum, Gibco, MA, Invitrogen), 50 nM ascorbate‐2‐phosphate (Sigma‐Aldrich, MO, USA), 0.1 mM dexamethasone (Sigma‐Aldrich, Saint Louis, MO, USA), and 10 ng/ml of human transforming growth factor (hTGF)‐β1 (Preprotech, UK) and seeded in GM10, or GM10 + BC, or GM10 + CS based biomaterials. Once the culture medium containing the cells was completely absorbed by the hydrogels, other 500 μl of medium were added to each well to cover the scaffold (matrix). The MSCs containing biomaterials were maintained at 37°C in a humidified atmosphere with 5% vol/vol CO_2_ until 21 days, replacing the culture medium every 48 h.

### 2.9 MTT assay

The ability of the cells to survive and proliferate on the cross‐linked scaffolds developed in this study was assessed after 7, 14, and 21 days of in vitro culture. Specifically, the tetrazolium dye 3‐(4,5‐dimethylthiazol‐2‐yl)‐2,5‐diphenyltetrazolium bromide (MTT) solution (0.5 mg/ml in DMEM nude) was added to the hydrogels in order to measure MTT reduction. After 3 hours, the insoluble purple formazan product was solubilized by hydrochloric acid (HCl) diluted in isopropanol. The optical densities of the obtained solutions were measured at 570 nm using a Beckman DU 640 spectrometer (Beckman, Milano, Italy). The relative cell viability was calculated as:

$$\text{Cell viability}\;\left(\%\right)=\frac{\left(\mathrm{OD}\;\mathrm{GM}10+\text{chondroitin}\;\right)}{\left(\mathrm{OD}\;\mathrm{GM}10-\text{chondroitin}\right)}\cdot 100\%.$$

Also, we reported the absorbance of each sample without normalizing to GM10. The experiments were performed in triplicate and the results showed as mean ± SD.

### 2.10 Gene expression of chondrogenic differentiation genes (qRT PCR)

After scaffold homogenization through a Tissue Ruptor Homogenizer (Qiagen, Hilden Germany), total RNA was isolated by TRIzol® Reagent (Invitrogen, Milan, Italy) as previously described.^39^ A Nanodrop Instrument (Celbio, Milan, Italy) was used to calculate the RNA concentration of each sample and 1 μg of total RNA was reversely transcribed into cDNA using the Reverse Transcription System Kit (Promega, Milan, Italy) following the manufacturer's instructions. Thus, a quantitative real‐time PCR (qRT PCR) was performed by the IQ™ SYBR® Green Supermix (Bio‐Rad Laboratories, Milan, Italy). All samples were analyzed in triplicate, and the mRNA expression of the following genes was normalized with respect to the glyceraldehyde‐3‐phosphate dehydrogenase (GAPDH) housekeeping gene: collagen type 1A1 (COLI), collagen type 2A1 (COLI II), SOX‐9 and aggrecan (AGN). The specific primer sequences used for the analyses are reported in Table 1. The variations of gene expressions were evaluated through Livak's method 2^‐ΔΔCt^ (ΔΔCt = difference of ΔCt between GM10 + BC or GM10 + CS and GM10) using Bio‐Rad iQ5 software (Bio‐Rad, Milan, Laboratories).^40^

**TABLE 1:** Primer sequences used for the qRT‐PCR

Gene	Forward primer	Reverse primer	AT PCR
GAPDH	5′‐TGCACCACCAACTGCTTAGC‐3′	5′‐GGCATGGACTGTGGTCATGAG‐3′	55°C
SOX‐9	5′‐AGACCTTTGGGATGCCTTAT‐3′	5′‐TAGCCTCCCTCACTCCAAGA‐3′	55°C
AGN	5′‐TCGAGGACAGCGAGGCC‐3′	5′‐TCGAGGGTGTAGCGTGTAGAG‐3′	55°C
COLI	5′‐CAGCCGCTTCACCTACAGC‐3′	5′‐TTTTGTATTCAATCACTGTCTTGCC‐3′	57°C
COLII	5′‐CAACACTGCCAACGTCCAGAT‐3′	5′‐CTGCTTCGTCCAGATAGGCAA‐3′	57°C

### 2.11 SEM analyses

For scanning electron microscopy, the samples were fixed in paraformaldehyde 4% vol/vol in PBS^−^, dehydrated in increasing ethanol percentage (ethanol 30–90% vol/vol for 5 min, absolute ethanol for 15 min for three times), dried in a critical point dryer and sputter coated with platinum‐palladium. After this preparation, samples were mounted on a stub and observed using a Supra 40 Zeiss scanning electron microscope.

### 2.12 Immunofluorescence for specific and not specific chondrogenic biomarkers

After 21 days of in vitro culture, the materials were fixed in a cold (4°C) solution composed of acetone: methanol (1:1 ratio) for 60 min at −20°C and then included in cryostat embedding medium (Bioptica, Italy) for cryosectioning and the sections were collected on slides. Antigen retrieval was obtained by incubation for 10 min at 95°C in tris‐EDTA buffer (10 mM Tris base, 1 mM EDTA solution, 0.05% Tween, pH 9). In order to permealize the samples; they were incubated with a blocking solution in a humid chamber. Primary antibodies against COLI (not‐specific chondrogenic biomarker) and COLII (specific chondrogenic biomarker) (diluted 1:100; Abcam, Cambridge MA), were incubated over‐night. Afterwards, the slices were washed in PBS^−^ and further incubated with FITC‐conjugated goat anti‐rabbit secondary antibodies (diluted 1:1000; Life Technologies, Milano, Italy) for 45 min. Cellular nuclei were stained with 2′‐(4‐hydroxyphenyl)‐5‐(4‐methyl‐1‐piperazinyl)‐2,5′‐bi‐1H‐benzimidazole trihydrochloride hydrate, bisBenzimide (Hoechst 0.5 mg/ml, Sigma‐Aldrich, Milano, Italy). Finally, the slices were sealed for observation; specific images were obtained by a fluorescence microscope Axiovert 200 (Zeiss) and analyzed through AxioVision 4.8.2. The mean pixel intensity for the labeled specific secondary antibodies was quantified using Quantity One 1‐D analysis software (Bio‐Rad, Hercules, CA, United States).

### 2.13 Histological analyses

Cryosectioned hydrogel samples, collected after 21 days of in vitro culture, were incubated at room temperature in Alcian blue solution (Sigma‐Aldrich; Merck, Darmstadt, Germany) for 5 min. The stained samples were then examined and photographed under a light microscope. Intensity of Alcian blue staining is directly correlated to the proteoglycan content.

## 3 STATISTICAL ANALYSES

Statistical significance of data was determined through one‐way ANOVA and Tukey post hoc test using JASP software (https://jasp-stats.org), in different experiments. In addition, two‐way ANOVA was performed (https://www.statskingdom.com/two-way-anova-calculator). Specifically, for the stability of the hydrogels toward hydrolysis and enzymatic degradation the results were reported as average of triplicates ± SD. The statistical significance was analyzed through one‐way ANOVA and Tukey post hoc test and two‐way ANOVA was performed considering as variables: the biomaterials formulation and the time. The outcomes of MTT assay were presented as average of triplicates and means ± SD, the statistical significance was analyzed through one‐way ANOVA and Tukey post hoc test, two‐way ANOVA was applied using as variables: the biomaterials formulation and the time. Moreover, the gene expression analyses were performed in triplicate and the relative the results shown as means ± SD. The statistical significance was analyzed through one‐way ANOVA and Tukey post hoc test and two‐way ANOVA was performed considering as variables: the biomaterials formulation and the gene expression. Finally, immunofluorescence staining of COLI and COLII in presence of GM10, GM10 + BC, and GM10 + CS was quantified through the mean pixel intensity of COLI and COLII staining. Data were expressed as means of three independent experiments ± SD. The statistical significance was analyzed through one‐way ANOVA and Tukey post hoc test and two‐way ANOVA was performed applying as variables: the biomaterials formulation and the protein expression.

## 4 RESULTS

### 4.1 Hydrogel preparation and physical characterization

As previously explained, the gelatin modification process was confirmed by ^1^H‐NMR spectroscopy and the resulting DM was 1.014 mmol/g. The complete spectra are reported in the supplementary materials (Figure S1).

**FIGURE 1:**
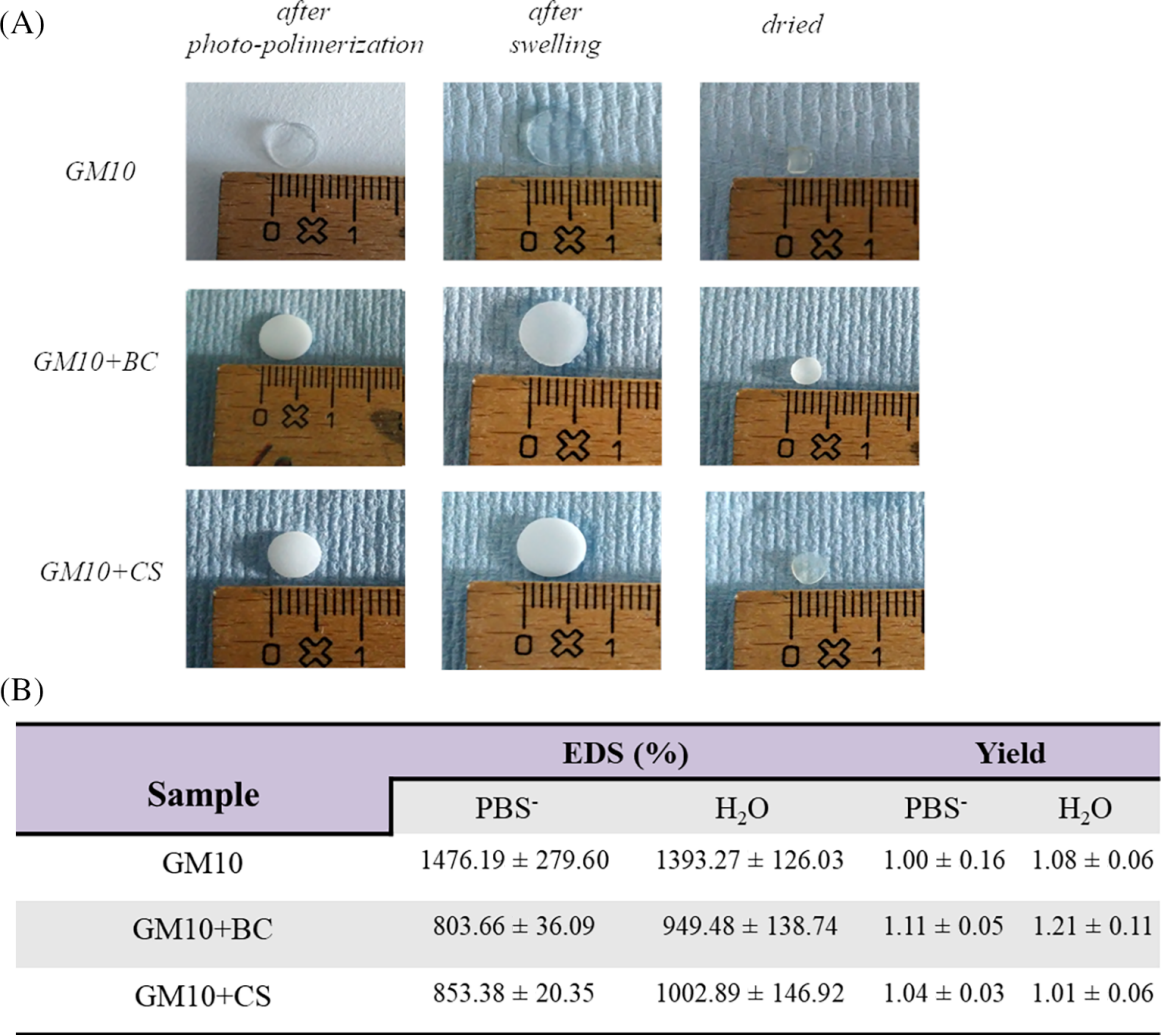
(A) Representative images of the hydrogels before, after the washing in PBS^−^ and finally dried. (B) EDS values (%) and yield for the hydrogels in PBS^−^ and H_2_O. EDS, equilibrium degrees of swelling

In all hydrogel preparation experiments, solid hydrogels were obtained after curing of the liquid precursor solutions. Figure 1A shows the appearance of the hydrogels before and after swelling in PBS^−^ (there were no observable differences for swelling in H_2_O). Whereas, the GM10 hydrogels were transparent, the hydrogels containing BC or CS were opaque.

The EDS of the biomaterials both in PBS^−^ and H_2_O are reported in Figure 1B. The EDS was evaluated after 3 days of washing at room temperature. The EDS of GM10 hydrogels was about 1476.19% in PBS^−^ and 1393.27% in H_2_O. The presence of BC and CS significantly (*p* < .05) reduced liquid up‐take ability in both experimental conditions, suggesting a more compact scaffold with hydrogen bonding already occurring between GM10 and the GAGs. Specific data for yield of each sample are reported in Figure 1B. The gel yields were generally quantitative. Additionally, HPCE analyses proved that only a small amount of BC and CS were released in PBS^−^ during 48 h of washing. The quantitative evaluation showed that about 10%–15% wt/wt of both BC and CS originally entrapped in the scaffolds were released from hydrogels within this time point. Relative data are reported in supplementary material (Figure S2). In addition, the cross‐linking of gelatin and the permanence of the two GAGs inside the hydrogels was confirmed also by RAMAN spectroscopy (data not shown).

**FIGURE 2:**
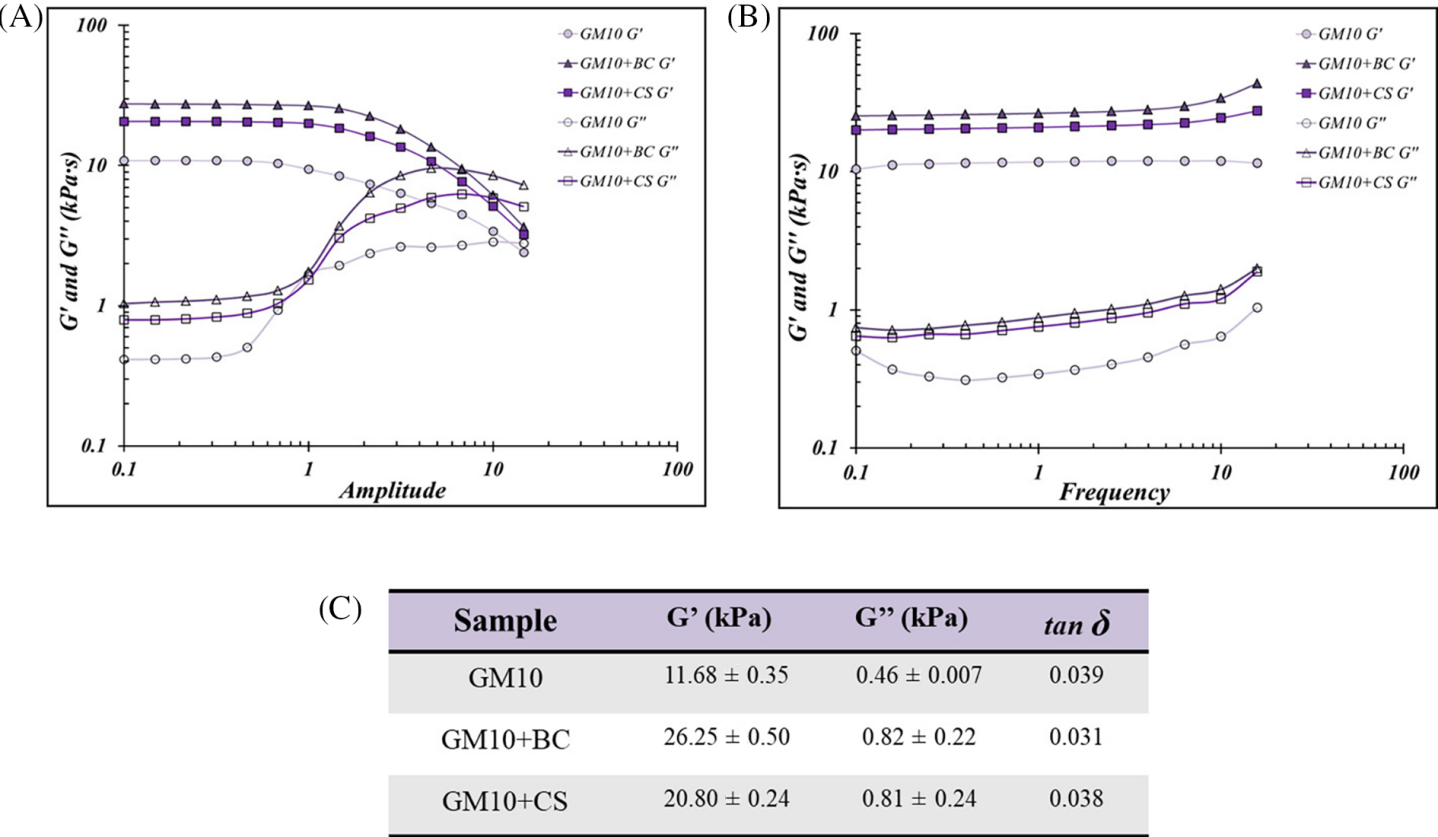
(A) Storage modulus *G'* and loss modulus *G″* for GM10 hydrogels with and without BC or CS measured as a function of amplitude. (B) *G′* and *G″* measured as function of the frequency. (C) *G′*, *G″*, and tan δ for each sample measured at frequency 0.7 Hz. Data are reported as average of three different preparations and means ± SD. BC, biotechnological chondroitin; CS, chondroitin sulfate

The differences in chemical composition of the hydrogels were also reflected in their rheological characterization. The amplitude sweep tests reveal that all the biomaterials had a *G'* significantly (*p* < .05) higher than *G″* at low amplitudes (Figure 2A) with a *G″/G′* ratio (*tan δ*) constantly << 1, again demonstrating the successful preparation of solid hydrogels. The results proved that BC and CS increased *G'* significantly (*p* < .05): for GM10 it was about 10.5 kPa while, for GM10 + BC and GM10 + CS it was about 26.9 and 20.5 kPa, respectively. Thus, the presence of chondroitins increased matrix rigidity in comparison to GM10 alone and this effect was more marked with BC (Figure 2A). In addition, *tan δ* was preserved constant among all the frequency range analyzed and also in this case, GM10 + BC and GM10 + CS presented a *G'* significantly (*p* < .05) higher with respect to GM10 (Figure 2). Specific values of *G'*, *G″* and *tan δ* measured at frequency 0.7 Hz are reported in the Figure 2C.

### 4.2 Stability of the hydrogels toward hydrolysis and enzymatic degradation

**FIGURE 3:**
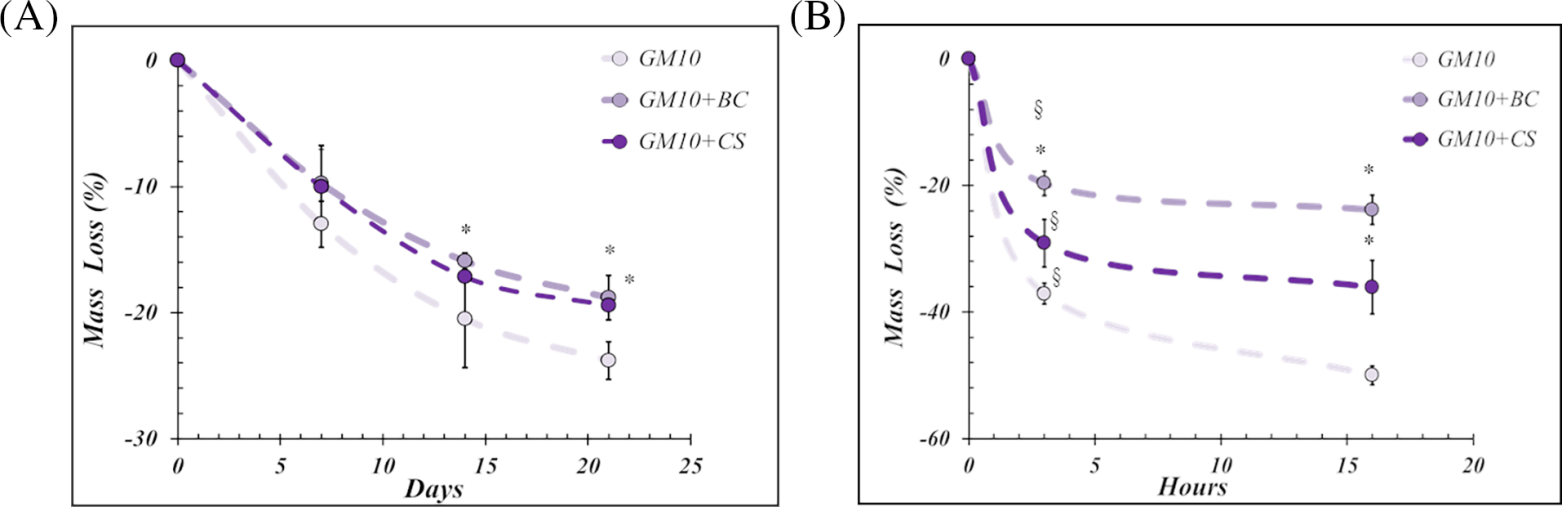
(A) The graphs show the mass loss (%) of hydrogels under physiological conditions after 7, 14, and 21 days and (B) incubated with a collagenase solution for 3 and 16 h. The results are reported as average of triplicates ± SD. The statistical significance was analyzed through one‐way ANOVA and Tukey post hoc test; *p < .05 versus GM10. JASP (https://jasp‐stats.org) was employed. Moreover, two‐way ANOVA was performed considering as variables: the biomaterials formulation and the time; §*p* < .05 versus the other scaffolds after 3 h (https://www.statskingdom.com/two‐way‐anova‐calculator)

The cross‐linked hydrogels seemed not varied in the appearance nevertheless, after 14 days of under physiological conditions incubation, GM10 + BC showed a significant (*p* < .05) minor mass loss in comparison to GM10. Moreover, after 21 days, in presence of both BC and CS the weight reduction was significantly (*p* < .05) lower than GM10 alone (Figure 3A). Furthermore, the addition of BC and CS to GM10 hydrogels significantly (*p* < .05) increased the biomaterials resistance to degradation when treated with collagenase (Figure 3B). In fact, after 3 h of enzymatic digestion, for GM10 hydrogels a mass loss of about 37% was detected while, in the presence of BC and CS this decrease was about 19% and 29%, respectively. After 16 h of incubation with collagenase, the mass loss for GM10 reached about 52%. Again, BC and CS based hydrogels were more resistant to enzymatic degradation with a mass decrease of about 23% and 32%, correspondingly.

### 4.3 MTT assay

**FIGURE 4:**
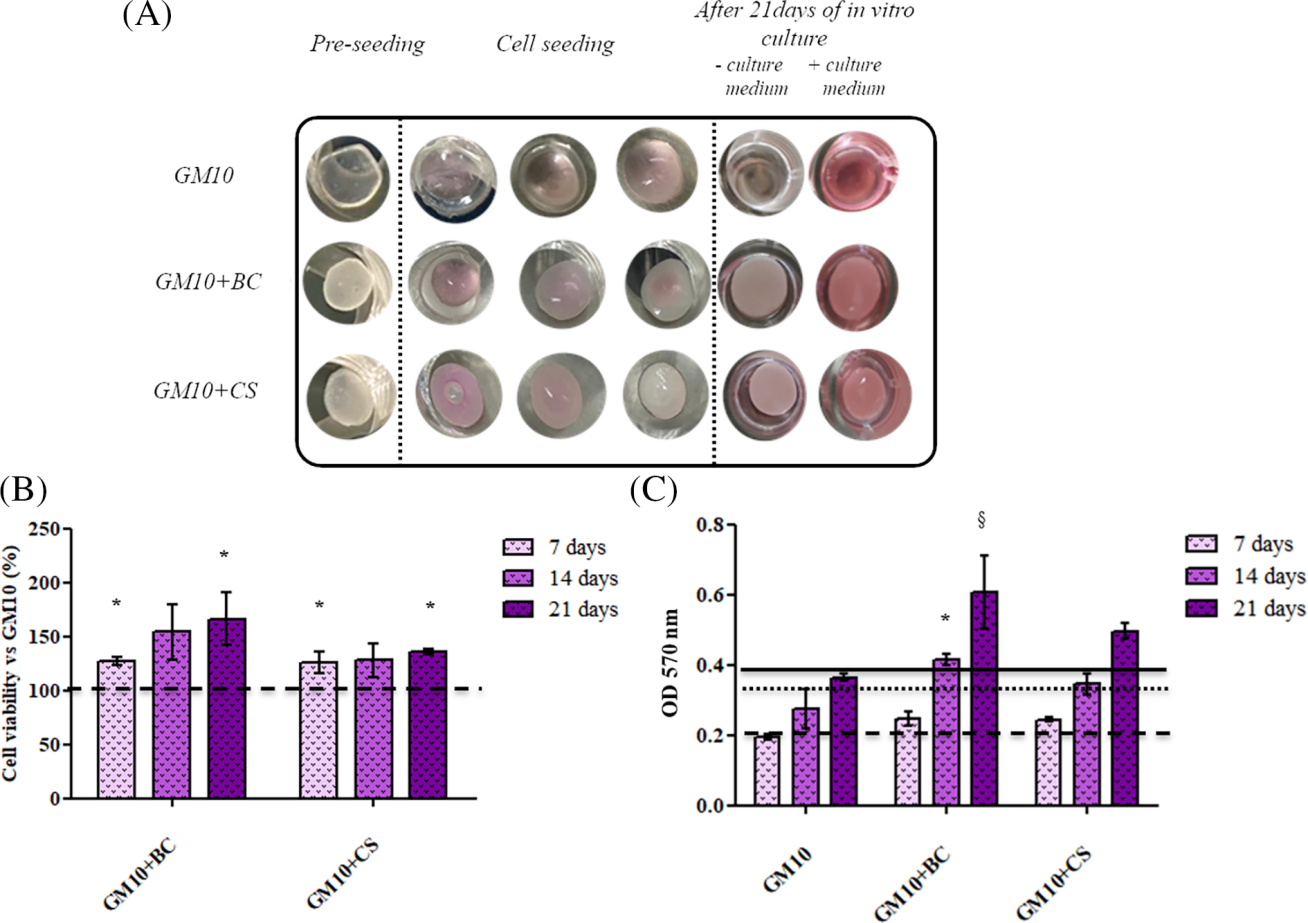
(A) Representative images of hydrogels in dried state, during MSCs seeding and after 21 days of in vitro culture. MTT results shown as average of triplicate and means ± SD, (B) Relative cell viability was calculated as following: vitality = 100 × mean OD GM10 + chondroitin /mean OD GM10‐chondroitin), (C) Absolute absorbance of each sample. The lines indicate the viability values of GM10 samples. The statistical significance was analyzed through one‐way ANOVA and Tukey post hoc test; *p < .05 versus GM10; #*p* < .05 versus GM10 + BC. JASP (https://jasp‐stats.org) was employed. Moreover, two‐way ANOVA was performed considering as variables: the biomaterials formulation and the time; §*p* < 0.05 versus the other scaffolds after 21 days (https://www.statskingdom.com/two‐way‐anova‐calculator). MSCs, mesenchymal stem cells

In order to evaluate the biological effects of cross‐linked scaffolds in terms of viability and proliferation of MSCs, MTT analyses were performed after 7, 14, and 21 days of in vitro culture. The results proved that none of the biomaterials was cytotoxic or dissolved itself among 21 days (Figure 4A). As shown in Figure 4B, after 7 days the presence of BC and CS significantly (*p* < .05) increased the cell proliferation with respect to GM10 alone (1.26‐ and 1.25‐fold, respectively, vs. GM10 hydrogel). Starting from 14 days of in vitro culture, GM10 + CS seemed not to further prompt the cellular proliferation. In contrast, GM10 + BC significantly improved MSCs growth and thus their vitality in comparison to GM10 (1.54 fold vs. GM10 hydrogel). Finally, after 21 days the significant (*p* < .05) increase of proliferation for the cells growth on GM10 + BC was markedly higher than GM10 + CS (1.66 and 1.36‐fold, respectively, vs. GM10 hydrogel) (Figure 4B). The Figure 4C reports the absolute absorbance at 570 nm of each sample without normalization. It is possible to note an increase of viability for all constructs in comparison to the sole GM10‐based scaffold.

### 4.4 Gene expression of chondrogenic differentiation genes (qRT PCR)

**FIGURE 5:**
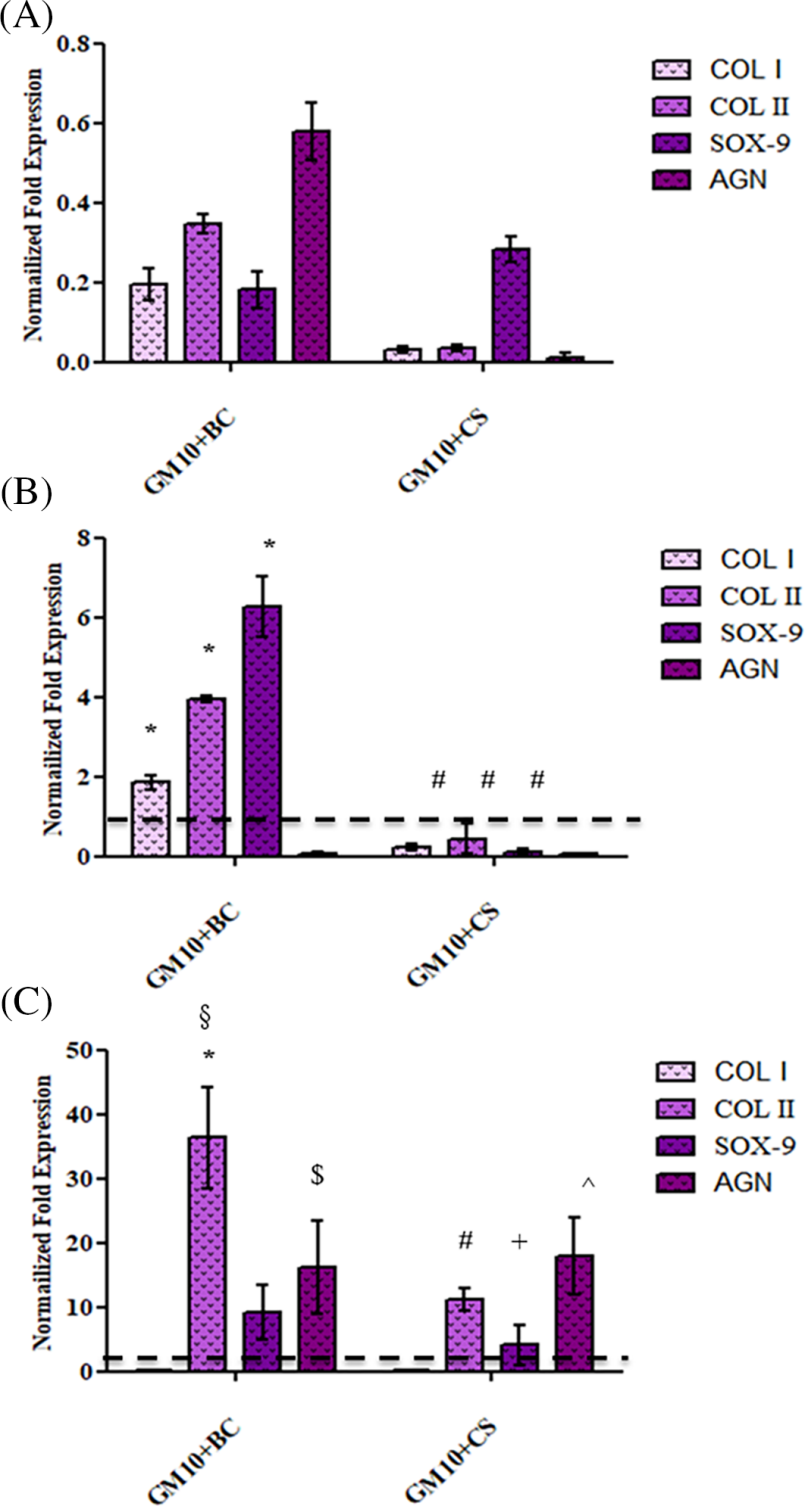
Quantitative gene expression analysis of COLI, COLII, SOX‐9, and AGN in MSCs after (A) 7 days, (B) 14 days, and (C) 21 days of in vitro culture with the hydrogels. The gene expression levels for GM10 + BC and GM10 + CS were normalized to GM10. The experiment was performed in triplicate and the results shown as means ± SD. The lines indicate the viability values of GM10 samples. The statistical significance was analyzed through one‐way ANOVA and Tukey post hoc test; *p < .05 versus GM10, #*p* < .05 versus GM10 + BC. JASP (https://jasp‐stats.org) was employed. Moreover, two‐way ANOVA was performed considering as variables: the biomaterials formulation and the gene expression; §*p* < .05 versus GM10 + BC after 7 and 14 days; ^*p* < .05 versus the GM10 + CS after 7 and 14 days; $*p* < .05 versus GM10 + BC after 7 and 14 days; + *p* < .05 versus GM10 + CS after 7 and 14 days (https://www.statskingdom.com/two‐way‐anova‐calculator). BC, biotechnological chondroitin; CS, chondroitin sulfate

To assess the effects of biomaterials on chondrogenic differentiation process, the gene expression of COLI, COLII, SOX‐9, and AGN was evaluated. Figure 5A shows that after 7 days of in vitro culture, all considered gene expression levels were down‐regulated in presence of BC and CS proving that the differentiation process did not begin. Instead, after 14 days (Figure 5B) COLII and SOX‐9 mRNA levels significantly (*p* < .05) increased 3.94‐ and 6.27‐fold with respect to GM10 for MSCs seeded in GM10 + BC, while GM10 + CS did not present a similar effect. In fact, at this experimental time‐point none of the analyzed genes for GM10 + CS resulted up‐regulated, thus highlighting an acceleration of differentiation process by BC. At longer time (21 days, Figure 5C), both GM10 + BC and GM10 + CS were able to improve the chondrogenic gene expression in comparison to GM10. In this context, BC was more effective than CS overexpressing COLII (*p* < .05) (36.30‐fold vs. GM10), SOX‐9 (9.20‐fold vs. GM10) and AGN (16.13‐fold vs. GM10). However, also CS up‐regulated mRNA expression of these genes with respect to GM10 (11.15 ‐fold, 4.10‐fold, and 17.94 respectively). In addition, it is interesting to note that COLI gene expression level, a specific biomarker of fibroblast phenotype, markedly decreased after 21 days of culture in both GM10 + BC and GM10 + CS while, after 14 days there was a slight overexpression compared to GM10 in presence of BC (Figure 5B).

### 4.5 SEM analyses

**FIGURE 6:**
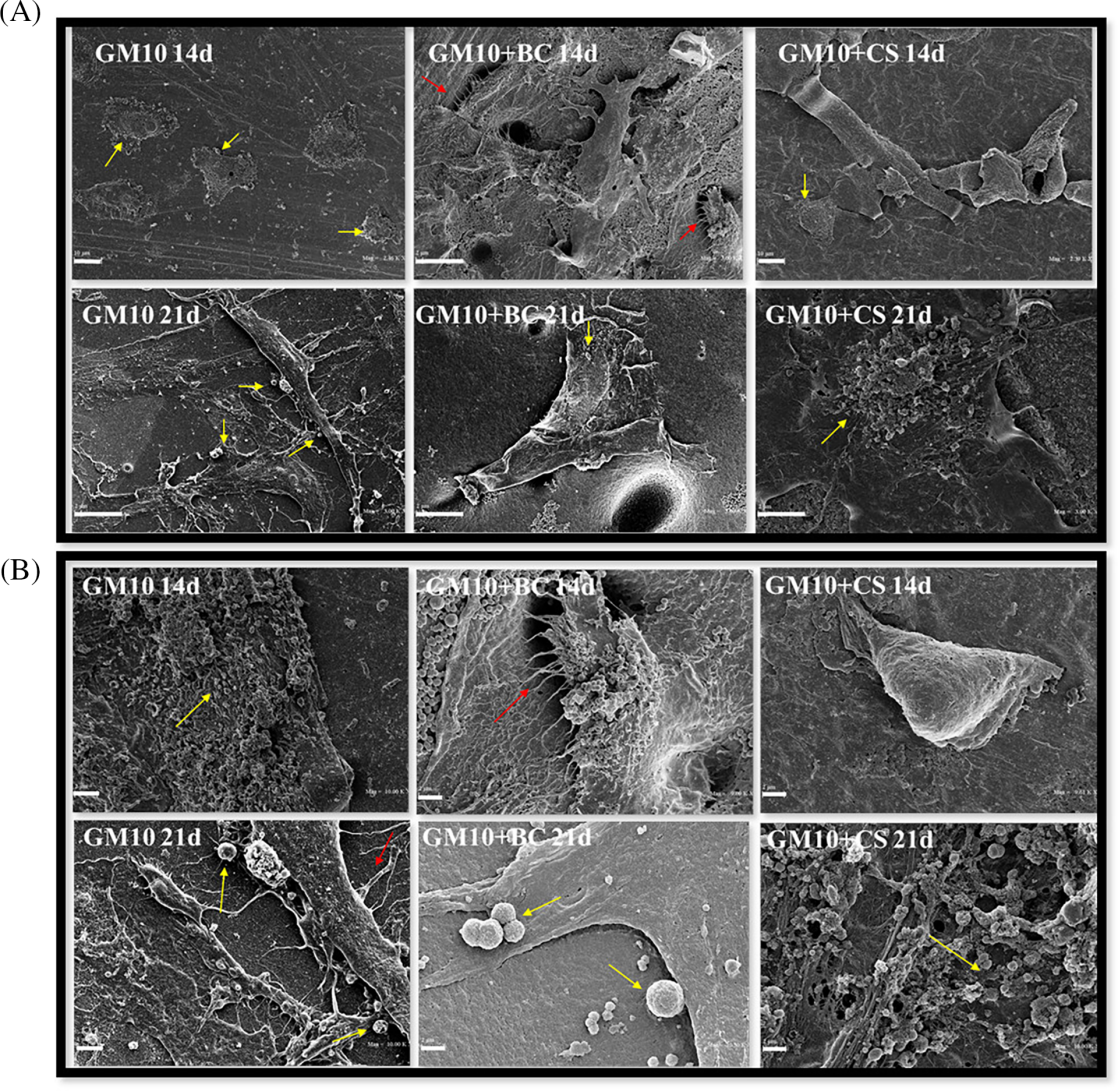
Scanning electron microscope observation of MSCs seeded on GM10, GM10 + BC, and GM10 + CS for 14 and 21 days. Red arrows indicate connection between cells and materials; yellow arrows indicate extracellular microvescicles. (A) Scale bar = 10 μm in low‐mag panel; (B) 2 μm in high‐mag panel. BC, biotechnological chondroitin; CS, chondroitin sulfate; MSCs, mesenchymal stem cells

In order to better characterize GM10 and GM10 + BC or GM10 + CS constructs, SEM observations were performed after 14 and 21 days of in vitro culture. As shown in Figure 6, the SEM analyses proved that MSCs adhere and grow on all materials, as confirmed by the formation of filopods (red arrows). Already at 14 days, the cells produced extracellular vesicles (yellow arrows); this phenomenon was even more evident at 21 days even if the distribution was different: more widespread on GM10 and GM10 + BC, more concentrated in one area on GM10 + CS. It can be noted that the cells grew forming clusters, but on GM10 they seemed more wrinkled while in the other cases they appeared smoother. Observation at higher magnification confirmed the production of extracellular vesicles. On average the vesicles formed by the cells seeded on GM10 + BC were larger (2 μm) than those on GM10 (lower than 1.5 μm). Finally, GM10 + CS, presented more heterogeneous vesicles dimensions, varying between 0.5 and 2 μm.

### 4.6 Immunofluorescence for specific and not specific chondrogenic biomarkers

**FIGURE 7:**
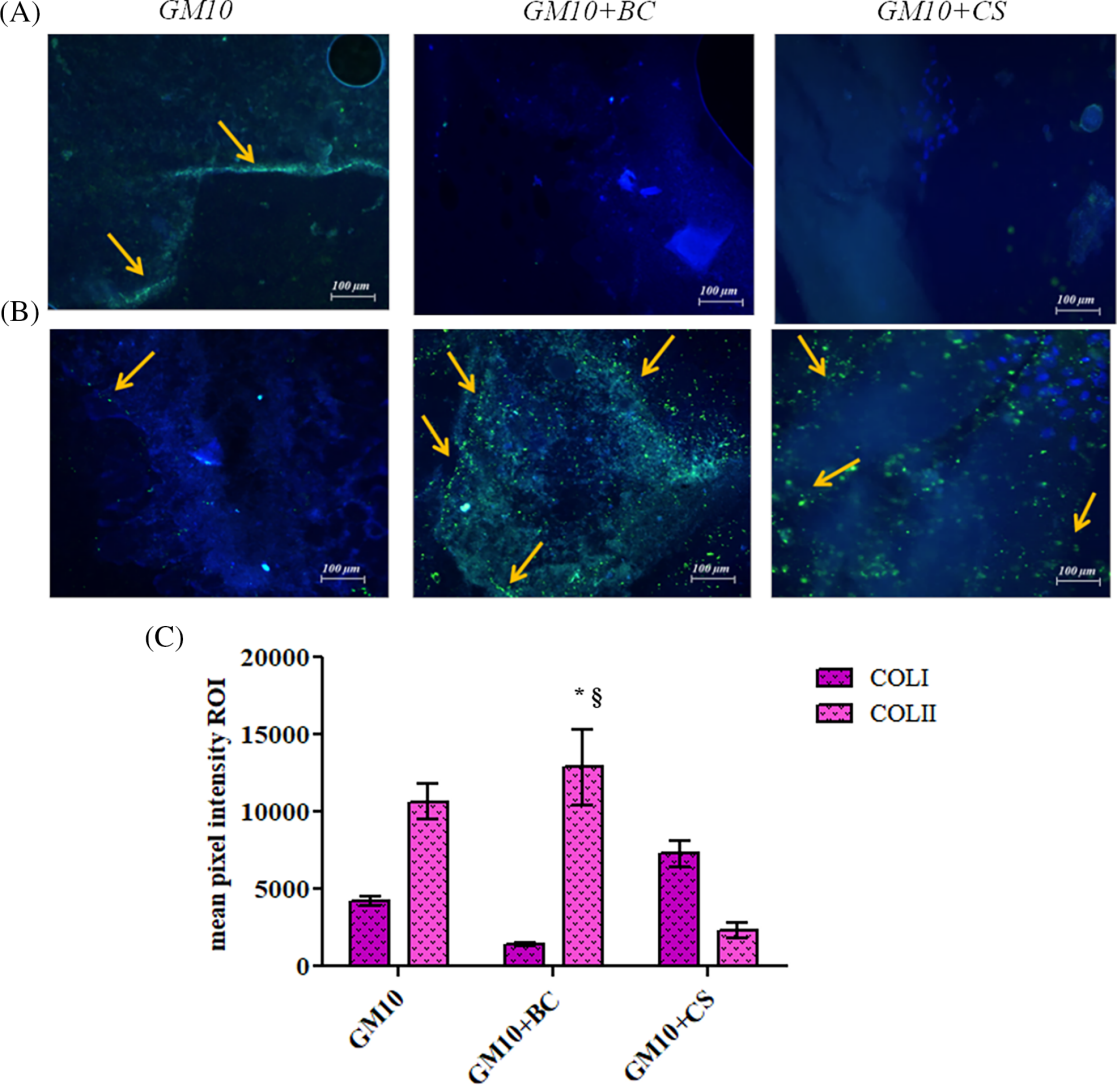
3D scaffolds after 21 days of in vitro culture with MSCs. Immunofluorescence staining of COLI (A) and COLII (B) in presence of GM10, GM10 + BC, and GM10 + CS. Blue nuclei, green COLI and or COLII. Microscopy magnification: ×20, yellow arrows indicate the cellular specific COLI or COLII signal. Graphs show the mean pixel intensity of COLI and COLII staining. Data are expressed as means of three independent experiments ± SD. The statistical significance was analyzed through one‐way ANOVA and Tukey post hoc test; *p < .05 versus GM10 and GM10 + CS (for COLII expression). JASP (https://jasp‐stats.org) was employed. Moreover, two‐way ANOVA was performed considering as variables: the biomaterials formulation and the protein expression; §*p* < .05 versus GM10 and GM10 + CS (for COLII). (https://www.statskingdom.com/two‐way‐anova‐calculator) (C). BC, biotechnological chondroitin; CS, chondroitin sulfate; MSCs, mesenchymal stem cells

Immunofluorescence staining revealed, after 21 days of in vitro culture, a consistent expression of COLII, as specific chondrogenic phenotype biomarker in chondroitin based samples. In fact, as shown by Figure 7B, in presence of BC and CS there was an evident accumulation of green signal compared to GM10 alone. Thus, this confirmed that both CS and BC increased COLII expression in MSCs. Moreover, COLI protein level was evaluated in all the biomaterials developed to verify if the cells were actually targeted toward the chondrocyte phenotype (Figure 7A). In fact, COLI is a fibroblast specific biomarker and the analyses proved that GM10 coupled to BC and CS reduced its expression as compared to COLII, supporting the ongoing chondrogenic differentiation process. As previous explained, the mean pixel intensity for the specific antibodies was quantified and the COLII production by GM10 + BC based scaffold resulted significantly (*p* < .05) higher than GM10 alone and GM10 + CS (Figure 7C).

### 4.7 Histological analyses

Histological analyses were inserted as Supplementary file (Figure S3) and showed the presence of different colored blue areas (yellow arrows). Also considering the outcomes of specific chondrogenic biomarkers expression analyses by qRT PCR and IF, these data could indicate a possible proteoglycan production by MSCs. Moreover, in order to confirm that the intensity of Alcian blue was prevalently due to the proteoglycan production by MSCs, also the materials without cells were stained. In this case the materials did not present relevant blue staining, thus the color revealed by seeded materials highlighted of the ability of MSCs to synthetize proteoglycans.

## 5 DISCUSSION

Nowadays, regeneration of damaged or defective cartilage is a field not yet fully explored by tissue engineering since current approaches are not able to completely regenerate the joint.^24^ Several studies are focused on developing innovative biomaterials able to support the MSCs chondrogenic differentiation process and characterized by mechanical properties miming the articular tissue complexity.^41^ In this context, because of its poor rheological properties, gelatin needs to be modified for application in cartilage regeneration.^6^, ^7^ Considering the previously well explored biological properties of CS and BC on several human primary in vitro cultures in terms of proliferation, anti‐inflammatory activity and tissue remodeling,^31^, ^33^, ^34^, ^35^, ^36^ the purpose of this experimental work was to evaluate their effect when combined to chemically modified gelatin. Firstly, the three obtained engineered hydrogels (GM10, GM10 + BC, and GM10 + CS) underwent mechanical characterization in order to verify the improvement of the viscoelastic behavior in presence of BC or CS. This characterization displayed a stiffer behavior in the presence of BC compared to GM10 + CS and GM10 hydrogels alone. Thus, GM10 + BC may constitute an interesting potential biomaterial for chondral tissue engineering since the articular cartilage needs strong viscoelastic properties in order to give resistance to compressive forces.^42^ The obtained data proved coherent with scientific literature, in fact, Gu et al. 2020 presented methacrylated gelatin scaffolds with the elastic moduli higher than loss moduli excluding a fluid‐like state^43^ and, also the rheological properties of GM10 biomaterials were similar to data present in literature.^43^, ^44^ Concomitantly, the introduction of BC and CS reduced the swelling. As recently shown, this effect may be ascribed to the semi‐interpenetrating structures and the reduction of ionic osmotic pressure in the presence of chondroitin.^18^ Moreover, the applied cross‐linking procedures resulted in materials not cytotoxic for MSCs. In fact, GM10 sustained the cellular viability, and the addition of BC and CS further increase proliferation in the first 14 days. In addition, the resulting biomaterials proved more stable under physiological conditions and more resistant to enzymatic degradation in comparison to GM10 alone. In this way, some of the most common obstacles to the use of gelatin in tissue engineering seem to be overcome.^6^, ^7^ These findings were coherent with scientific literature, in fact, it is reported that combining CS to gelatin improved resistance to collagenase degradation and storage modulus.^4^ Another important prerequisite for a biomaterial, to be considered suitable and performant in tissue engineering application, is to support cell adhesion, proliferation and facilitate the nutrients transport.^45^ All the hydrogels here tested confirmed the capacity to support MSC viability, in addition, chondroitin, in particular BC, prompted the chondrogenic differentiation already after 14 days of in vitro culture. These results are coherent with the recently reported finding that showed CS and even more BC (combined to HA) may be used in MSC media to obtain higher cell viability, lower senescence and more extensive differentiation.^36^ In detail, MSCs differentiation is a multi‐step process regulated by a sequence of different signaling pathways and growth factors. In this context, several scientific studies revealed that MSC‐derived small extracellular vesicles (MSC‐EVs) may enhance the regenerative capacity of MSCs.^46^ Generally, the EVs include exosomes (with a diameter of 30–120 nm) derived from the endocytic pathway, small and heterogeneous microvesicles (100 nm–1 μm) originating from membrane budding and also larger vesicles (>1 μm).^47^ MSCs‐EVs may contain proteins, lipids, mRNA, microRNA and they are involved in cell communication, transcription modulation, cell survival, and differentiation. For these reasons, EVs are proposed as a new tool in regenerative medicine studies.^48^, ^49^ Thus, in this experimental set‐up, the superior biological effectiveness of GM10 + BC and GM10 + CS in comparison to GM10 may be related to a more evident accumulation of MSCs‐EVs as shown by SEM observations. Furthermore, it is interesting to note, that the cells were able to express COLII and this is a differentiation marker toward chondrocytes phenotype, even if a fully mature tissue was not yet formed, at 21 days incubation, as demonstrated by the Alcian blue staining. Finally, our outcomes suggest that both BC and CS maintain their biological efficacy when coupled to GM10 and exposed to hydrogel cross‐linking procedures. Specifically, BC, for the first time tested as component of this kind of scaffolds, resulted more performant than CS in improving biophysical parameters and supporting MSC differentiation processes. Thus, the mechanical and swelling features, with high‐cell survival and differentiation toward the chondrocyte phenotype, support the developed biomaterials as potential candidate materials for the management of cartilage regeneration. However, it is important to point out that 3D scaffolds were obtained but their biological potential should be further explored in the future. Specifically, tuning porosity and also increasing the initial seeding cell density may be of interest to permit/improve extensive/complete colonization of the biomaterials. Overall, given that, BC leads a differential biological response, the specific cellular mechanism activation should be further investigated. An hypothesis may be related to the BC similarity to the only unsulfated GAGs naturally occurring, namely HA. In this respect, a binding to CD44 receptor may be expected; however, this has to be ascertained as the size of the two molecules differ for 2 order of magnitude. Nonetheless, BC displays acetylated glucosamine, that is present in CS thus, also the more specific sulfate chondroitin receptors involvement may be considered.^50^

## 6 CONCLUSIONS

This research aimed to compare the biophysical properties and biological effects in chondrogenic differentiation process of three different cross‐linked hydrogels based on chemically modified gelatin alone and coupled to BC or CS. In this respect, for the first time, a biofermentative unsulfated chondroitin was tested in a newly synthetized chemically cross‐linked gelatin based matrix, proving effective as potential scaffold with applications in the field of cartilage regeneration. The results demonstrated that the presence of CS and mostly of BC improved mechanical features of GM10 based hydrogels and prompted the differentiation of MSCs through the over‐expression of specific genes. A potential role of extracellular vescicles is also suggested. Further experiments are needed for a full translational perspective on the application in the management of damage and/or loss cartilage of the developed hydrogels based on innovative GAGs here presented.

## CONFLICT OF INTERESTS

The authors declare that there is no conflict of interests.

## AUTHOR CONTRIBUTIONS

Chiara Schiraldi and Günter E.M. Tovar conceived and designed the experimental work. Chiara Schiraldi, Günter E.M. Tovar, Alexander Southan, and Valentina Vassallo interpreted the obtained data. Anastasia Tsianaka and Jana Grübel chemically modified the gelatin and analyzed its modification degree. Valentina Vassallo and Alexander Southan run the biophysical analyses on gelatin‐based scaffolds. Valentina Vassallo performed the biological tests, Nicola Alessio carried out the histological characterization and Marcella Cammarota performed SEM investigation. Chiara Schiraldi, Günter E.M. Tovar, and Alexander Southan revised and edited the manuscript. All the coauthors contributed to the assessment of the results and agreed to the published version of the manuscript.

## Supporting information


**Supplementary file S1** ^1^H‐NMR analyses results of modified gelatin

**Supplementary file S2** HPCE analyses results of PBS‐hydrogels washing among 48 h

**Supplementary file S3** Supporting information

## Data Availability

The data are reported in the results session and in supplementary files, any other request of data can be addressed to correspodnding authors that will share the specific files or the original images.
