# Supplementary figures and images for: Evaluation of novel biomaterials for cartilage regeneration based on gelatin methacryloyl interpenetrated with extractive chondroitin sulfate or unsulfated biotechnological chondroitin
Source: J Biomed Mater Res A. 2022 Jan 28;110(6):1210–23. doi: 10.1002/jbm.a.37364 (PMC9306773; doi:10.1002/jbm.a.37364)

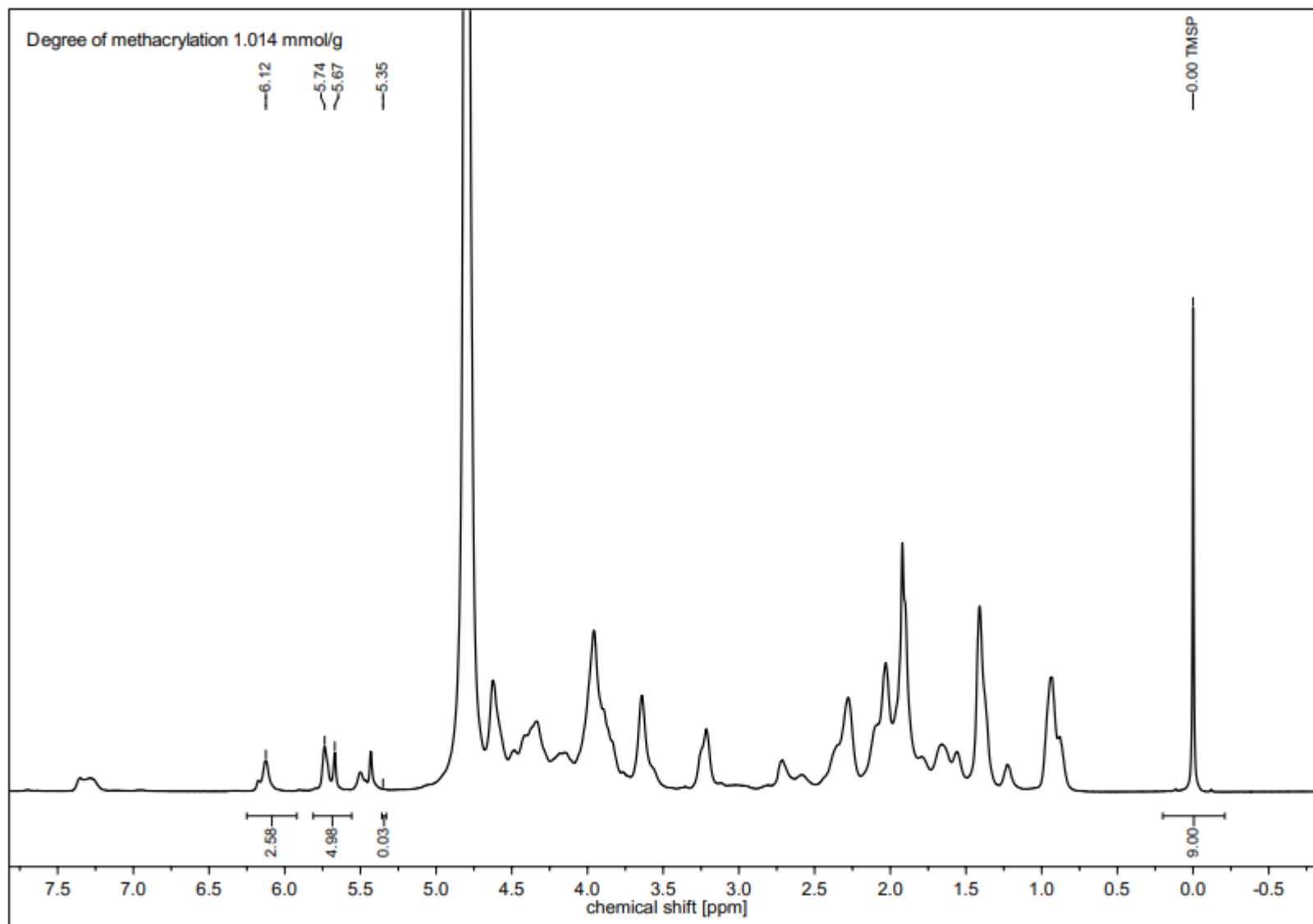

Supplementary file (S1).  $^1\text{H}$ -NMR analyses results of modified gelatin.

Supplement: Supplementary file 1 — Supplementary file S1 1H‐NMR analyses results of modified gelatin [file JBM-110-1210-s002.pdf]

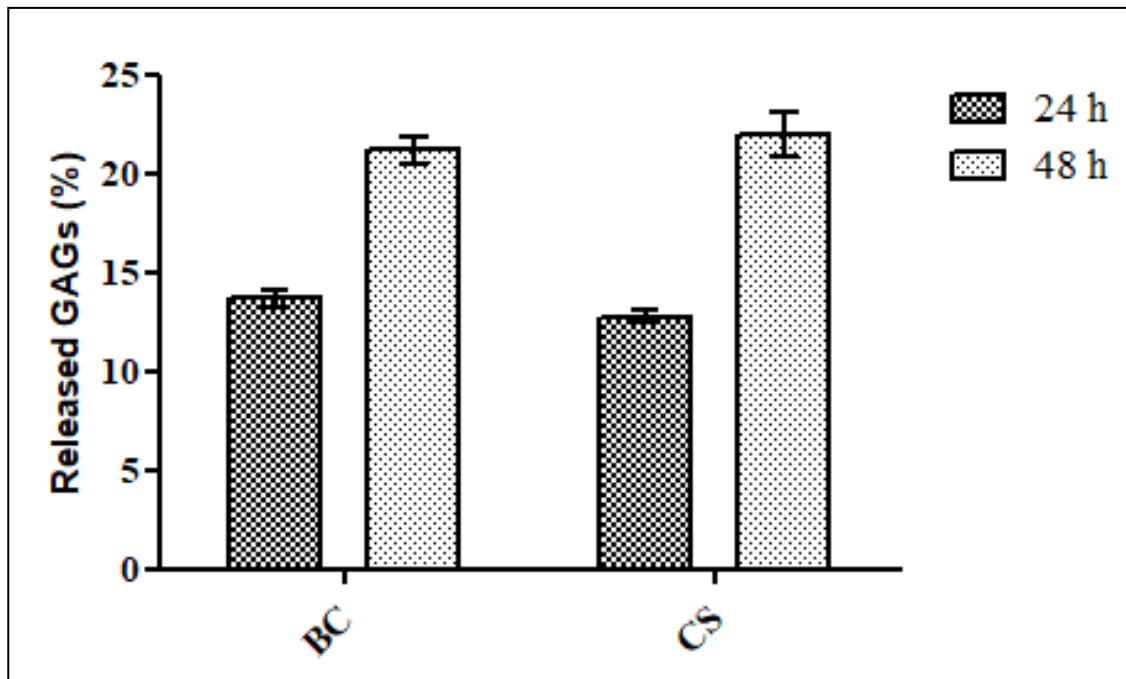

Supplementary file (S2). HPCE analyses results of PBS<sup>-</sup> hydrogels washing among 48 hours.

Supplement: Supplementary file 2 — Supplementary file S2 HPCE analyses results of PBS‐hydrogels washing among 48 h [file JBM-110-1210-s001.pdf]

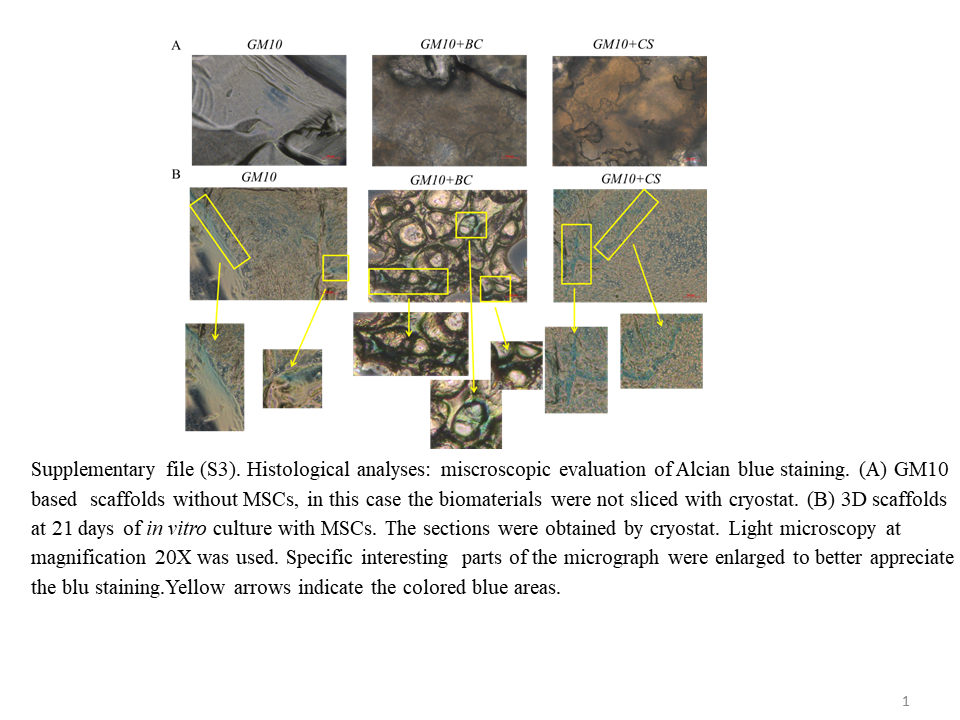

Supplement: Supplementary file 3 — Supplementary file S3 Supporting information [file JBM-110-1210-s003.tif]
